# Resilience: A panacea for burnout in medical students during clinical training?: A narrative review

**DOI:** 10.1097/MD.0000000000040794

**Published:** 2024-12-06

**Authors:** Ardo Sanjaya, Nathanael Andry Mianto, Karen Regina Wijayanto, Christian Edwin

**Affiliations:** a Department of Anatomy, Faculty of Medicine, Maranatha Christian University, Bandung, Indonesia; b Biomedical Research Laboratory, Faculty of Medicine, Maranatha Christian University, Bandung, Indonesia; c Department of Pulmonology, Faculty of Medicine, Maranatha Christian University, Bandung, Indonesia; d Undergraduate Program in Medicine, Faculty of Medicine, Maranatha Christian University, Bandung, Indonesia; e Medical Education Unit, Faculty of Medicine, Maranatha Christian University, Bandung, Indonesia.

**Keywords:** burnout, clinical clerkship, clinical competence, medical students, resilience

## Abstract

Clinical rotations or clerkships are a necessary part of medical education but are associated with significant psychiatric morbidity, including burnout and psychological distress. This review aims to explore the role of resilience as a protective factor and assess the effectiveness of resilience-building interventions. We conducted a narrative review using the Medline database via PubMed. The search strategy included the terms “resilience,” “burnout,” and “medical students.” A total of 52 articles were included and synthesized narratively. Burnout affects 40% to 80% of medical students, with higher rates during their clinical years. Resilience was consistently found to be a stable, protective factor that buffers against stress and burnout. Most research focused on medical students, regardless of whether they are in clinical training. However, the effectiveness of resilience interventions varied. Interventions such as psychological workshops and curriculum changes showed mixed results, with competence-based approaches showing the most promise for long-term benefits. Resilience is a critical target for improving medical students’ psychological health and reducing burnout during clinical training. Future interventions should focus on combining psychological support with competence-based training to equip students for the challenges ahead.

## 1. Introduction

Clinical rotations or clerkships are a necessary part of medical education. Although essential, this part of education is associated with significant psychiatric morbidity. Research has shown that clerkships were associated with burnout, depression, anxiety, and psychological distress.^[1,2]^ These burdens present additional difficulties for students who already face a significant transition of learning experience from the theory-based preclinical years to the active learning experience of clerkships. As educators, we must prepare students for their clinical practice and protect them during training.^[3]^ Additionally, these difficulties faced during training, in turn, may hamper learning. Interventions directed at those difficulties may help students adjust better and increase performance.

Burnout is one of the most measured psychological distresses in medical students and practicing physicians. Burnout is a stress syndrome from chronic exposure to job stress.^[4]^ And is usually measured using self-reported questionnaires. Although a subjective measurement, burnout is associated with significant learning challenges and clinical interactions. Several studies found that burnout affects at least 40% of medical students.^[5–8]^ Students in their clinical years had a higher prevalence of burnout than students in the lower academic year.^[9,10]^ Interestingly, early in their clinical years, these students have the same mental health level as the general population,^[11]^ signifying that the stresses of clinical rotation are a significant factor contributing to the decrease in mental health in these students. Several studies have found that burnout among medical students is higher than the general population, even accounting for significant adverse childhood events.^[12–15]^ Making it even worse, a study found that protective factors for mental health in students were impaired after a little more than the first semester of study.^[16]^ These previous findings have shown that the rigors of training might be detrimental to their psychological health and are, therefore, an intervention target.

The transition to clinical years has been well-defined as a source of stress. Several studies reported that some of the common problems stated by clinical students were personal well-being and interpersonal issues.^[17–21]^ Several factors played a role in developing significant psychiatric morbidity and psychological distress during clerkships. These factors were, but not limited to, professionalism dilemmas,^[17]^ inadequate coping strategies,^[22]^ increased workload with less time for studying,^[1,18]^ and previous psychiatric conditions. Most of the factors mentioned are unique to clerkships and present significant obstacles that students must face during their medical training. However, preparing students for the multitude of experiences that they can face during clerkships may be impractical due to the unpredictability of the problems that might occur.^[23]^ A more feasible approach may rely on equipping students with the necessary coping skills to face real-world dilemmas and difficulties encountered in clinical and educational settings. One such construct that may be applicable in this situation is resilience.

Resilience has been defined in many ways, each focusing on it as a process, a trajectory, an end goal, and many other fields.^[24]^ Resilience can be summarized as overcoming difficulties with perseverance and thriving amidst those difficulties.^[25,26]^ Several studies have shown that resilience is a protective factor associated with better student perception and quality of life.^[27–29]^ Additionally, resilience was stable over time and may be a better target than the myriad of distress students face during their clinical training.^[23]^ Although usually considered a psychological construct, resilience consists of many domains. It can be influenced through competency training and indirectly through the hidden curriculum in the medical school.^[24,30,31]^

This review addresses these gaps to support students in high-stress conditions by exploring resilience as a protective factor against burnout and stress and the effects of various resilience-building interventions. We hope our results will help provide recommendations for resilience training in the medical school’s curriculum and enhance student well-being and performance.

## 2. Materials and methods

A general search strategy using the Medline database through PubMed was conducted to conduct this narrative review. The search strategy was “resilience, psychological” [MeSH Terms] AND “students, medical” [MeSH Terms] AND “burnout, professional” [MeSH Terms]. However, our initial search strategy only resulted in 25 articles. Therefore, we refined our search strategy only to include “resilience, psychological” [MeSH Terms] AND “students, medical” [MeSH Terms]. This strategy resulted in 135 articles further screened by 2 authors independently. The eligibility criteria used by the authors for screening are in Table 1. After the full-text screening, 52 articles were included in our review (Table S1, Supplemental Digital Content, http://links.lww.com/MD/O109). The articles will be summarized and synthesized narratively. No ethical approval is sought due to the nature of this study.

**Table 1:** The eligibility criteria of articles included in the study.

Inclusion criteria	Exclusion criteria
Studies focusing on medical students.	Articles not in English.
Studies discussing any measures of resilience, burnout, or stress.	Opinion pieces, editorials, or other non-peer-reviewed sources.
Cross-sectional, cohort, interventional, and meta-analysis of trials.	

## 3. Results

Fifty-two articles were synthesized in our current review of resilience and its effects on medical students. These articles encompass various study designs from cross-sectional surveys, prospective cohort studies, and interventional studies. In this research, we will synthesize the previous articles in the context of resilience, its buffering effects on burnout or psychological stress, and the effectiveness of resilience-building interventions. We identified several recurring themes from the articles that we have extracted. The complete themes and articles in our analysis are in Table S1, Supplemental Digital Content, http://links.lww.com/MD/O109.

### 3.1. The scope of the problem: the prevalence and impact of burnout in medical student

Burnout is one of the most measured psychological distresses in medical students and practicing physicians. Burnout is a stress syndrome from chronic exposure to job stress.^[4]^ And is usually measured using self-reported questionnaires. Several studies found that burnout affects 40% to 80% of medical students, with rates dependent on the study design and country.^[5–8]^ Students in their clinical years had higher levels of burnout than students in the lower academic year, with scores of exhaustion and depersonalization increased by up to 5 points (22.3 vs 25.0 and 10.0 vs 15.1, respectively).^[9,10]^ Interestingly, early in their clinical years, these students have the same level of mental health compared to the general population,^[11]^ signifying that the stresses of clinical rotation are a significant factor in contributing to the decrease in mental health in these students. Several studies have found that medical students’ burnout is higher than the general population, even accounting for significant adverse childhood events. Making it even worse, a study found that protective factors for mental health in students were impaired after a little more than the first semester of study (average difference of resilience score -2.44; *P* < .01) compared to the general population.^[16]^ These previous findings have shown that the rigors of training might be detrimental to their psychological health and are, therefore, an intervention target.

Several problems are readily apparent with the high prevalence of burnout. One such issue is how burnout detrimentally affects learning. Although we can assume that psychological stress is associated with worse learning outcomes, several studies support that notion. Burnout is associated with lower learning engagement during medical school.^[32]^ Other studies have found that burnout affected students’ perception of their learning environment,^[33]^ with those with higher burnout scores associated with worse learning environment perception.^[34]^ Those with higher learning environment scores were associated with higher academic achievement.^[35]^ Additionally, those who perceive their learning environments less favorably are more likely to regret choosing a medical career and are more likely to be exhausted, disengaged, and have lower levels of empathy.^[36,37]^

This article focuses more on students undergoing clinical training or clerkships. Students describe this transition as abrupt and creating the feeling of incompetence and impostor syndrome.^[18]^ This feeling of incompetence was associated with a higher prevalence of burnout.^[8]^ Furthermore, unique during clerkships, medical students face challenges such as, but not limited to, traumatic experiences and interpersonal problem,^[17,38]^ aside from the increased workload and burden of working and studying in healthcare settings.^[1,18,39]^ These challenges were shown to positively influence personal growth at the cost of increased risk of depression.^[38]^ Although personal growth might initially seem beneficial, an author identified that medical students in their clerkship years were more likely to develop adaptive and maladaptive coping skills.^[40]^ They were making this growth more of a haphazard and random byproduct of clinical education. Additionally, the clinical years were shown to be less supportive of students’ basic psychological needs, namely autonomy, and therefore present additional challenges.^[41]^

Another unfavorable outcome associated with burnout and stress during clinical training is the increased prevalence of errors and decreased professionalism among students.^[37,42]^ Although the evidence of medical errors in students is scarce, several studies on physicians have associated burnout with increased patient safety incidence^[37]^ and were also associated with an increased probability of self-reported errors.^[42]^ These errors were independently associated with emotional exhaustion, one of the core components of burnout. Adding to the evidence for the unfavorable outcome of burnout, emotional exhaustion is associated with low competence and low resilience among trained physicians.^[43]^ Resilience might be the answer we need to protect our future physicians currently in training.

### 3.2. Resilience as a buffer for burnout

Although much research describes the relationship between resilience and stress in medical students, it is challenging to synthesize and compare them due to the myriads of definitions regarding resilience. Resilience was found to mediate the effects of known protective factors and buffer the impact of stress on medical students.^[44]^ Additionally, research has found that resilience is a stable trait over time and is, therefore, a promising target for improving student’s mental health.^[23]^ Therefore, no available, high-quality data supports the causal relationship between high resilience and better student mental health outcomes. Nevertheless, there are many studies linking resilience and student’s mental health.

Cross-sectional studies conducted in Brazil, South Korea, and India have shown a negative correlation between resilience and burnout.^[45–48]^ A prospective study by Lin et al, which followed students for a year, identified resilience as a buffer between stress and burnout. Their findings were confirmed by Yu et al, who corroborated resilience as a factor that mediates burnout and medical students’ well-being. Several other authors’ research consistently proved these findings.^[28,41,47,49–52]^ Although no research specifically evaluated resilience’s ability to buffer stress in clinical settings, the findings found by medical students should be directly translatable to clerkships.

### 3.3. Effects of resilience-building interventions

Our review identified several studies that aimed to teach resilience to medical students. A survey by Houpy et al identified that the students themselves admitted to the need for resilience training, especially during clinical training.^[53]^ Several studies have tried resilience training as workshops and seminars^[54–62]^ or even through incorporating them into the curriculum.^[63–65]^ The results of these studies varied considerably (Table 2), ranging from no effect to a small-moderate development. In this review, we would like to examine several novel interventions proposed by these authors.

**Table 2:** Summary of resilience-building interventions in the review.

Intervention	Study author	Study design	Sample characteristics	Key findings
Psychological Interventions for Resilience	Kunzler et al (2020)^[66]^	Cochrane Systematic Review	30 studies, 19 analyzed in the meta-analysis	There is a low level of evidence that resilience training improves resilience and symptoms of anxiety and depression.
Emotional Speed Dating	Kiesewetter and Dimke (2020)^[53]^	Intervention study	Unknown	Increased emotional regulation; positive feedback from participants.
Shame Resilience Seminars	Bynum et al (2019)^[54,55]^	Pre-post-Intervention	113 second-year medical students	Improved handling of shame and reduced social isolation.
Training for Awareness Resilience and Action (TARA)	Ekbäck et al (2022)^[58]^	Single-arm, Pre-post Intervention	23 early semester students	No significant improvement in resilience scores; minor improvement in depression and anxiety.
Active Resilience Training (Adapted US Military Program)	Mugford et al (2022)^[56]^	Prospective Cohort	35 students	Skills retained for 18 months; increased stress management ability.
Wellness Curriculum Integration	Edmonds et al (2023)^[62]^	Curriculum change	58 students	Increased well-being, reduced anxiety and depression.
Clinical Competence: “Hemorrhage Control Training”	Levy-Carrick et al (2019)^[64]^	Single-Arm, Pre-Post Intervention	126 students	Increased resilience-associated traits (e.g., empowerment, self-efficacy, confidence).
Team Debriefing After Clinical Events	Martinchek et al (2017)^[61]^	Pre-post intervention	30 students	No change in resilience scores post-intervention.
Online Resilience Programs	Ungar et al (2022)^[67]^	Systematic Review	11 articles	There is very limited evidence of the effectiveness due to the majority (9) lacking controls.

Several approaches to resilience training were conducted by these authors, ranging from psychological interventions to other less-studied approaches, such as curricular changes and competence training. A Cochrane review on the effectiveness of psychological interventions for resilience training on healthcare students found very low-quality evidence for the effect of resilience training on stress, anxiety, and stress perception of students.^[66]^ The authors note that the main problems were the intervention heterogeneity and the absence of long-term follow-up data. Additionally, all studies in the review use various psychological interventions ranging from educative seminars, eye movement desensitization therapy, stress management, and neurolinguistic programming.

An innovative approach to resilience training is conducted by Kiesewetter and Dimke using the principle of speed dating, dubbed “Emotional speed-dating.”^[56]^ Students were paired and took turns interviewing for 3 minutes. The author argued that these experiences allow students to normalize handling negative emotions and provide an outlet for students experiencing frustration and helplessness. Other authors approach resilience with a more conventional intervention. Through addressing shame using seminars and workshops that teach students how to identify and constructively handle shame.^[57,58]^ However, their research did not directly measure its effects on resilience; additionally, since the study uses a pre-post design, no data is available on student retention of those skills. A study conducted in Sweden found that their specific intervention is called Training for Awareness, Resilience, and Action.^[61]^ The authors of the study concluded that the Training for Awareness, Resilience, and Action intervention improved the scores on depression, anxiety, stress, and psychological inflexibility, although no statistically significant difference was observed. However, their study did not have a control arm and did not measure whether their intervention influenced resilience. Two studies approached resilience similarly through workshops focusing on resilience or structured team debriefings.^[63,64]^ However, both studies did not thoroughly assess their effects on resilience after the intervention. The study by Bird et al evaluated resilience using the Connor–Davidson Resilience Scale, yet its use is optional, with no data on the survey’s response rate.^[63]^ Additionally, only 1 of the 2 centers showed significant changes in the resilience score, further complicating interpretation. The study by Martinchek et al did not report any resilience measures after intervention, although Connor–Davidson Resilience Scale distribution was part of their intervention.^[64]^

These previous studies did not validate whether their intervention is effective at increasing resilience or has a short follow-up period. Therefore, there is no data on whether these changes are retained after several months. Several studies have attempted to answer these drawbacks. A study by Mugford et al utilized a course called Active Resilience Training, adapted from those used by the United States Military. They reported that students continue to utilize the knowledge gained from the “Active Resilience Training” program used in the study for at least 18 months after the end of their training.^[59]^ Another study tried delivering resilience training for medical students in 1 year. The authors did not find any significant improvement in burnout and resilience scores. However, scores measuring happiness and quality of life decreased while those measuring stress increased. This reflects that delivering mandatory interventions over a long span did not directly translate to improvements and may be deleterious.^[62]^ Additionally, short online programs offering resilience training were shown to be effective while providing these students flexibility, which might be a better option.^[67]^

Another innovative approach to increasing resilience did not involve teaching coping skills or another measure of psychological health. A study by Brennan et al focused instead on addressing the students’ clinical experience. They identified that the stress and demand of the transition to clinical years can be reduced by increasing student’s clinical experience during their undergraduate years.^[68]^ This experience is better learned through early exposure to real-world encounters than simulated patients.^[69]^ Similar to their approach, another group focused on student competence instead of other psychological factors. They identified that students with a sense of satisfaction regarding their competence were likelier to have increased resilience scores.^[30]^ Proving their findings, an experiment conducted by Levy-Carrick et al found that training for clinical competence with their “Hemorrhage Control Training” increased resilience-associated traits such as empowerment, self-efficacy, and confidence in medical students.^[70]^ These findings suggest that aside from psychological interventions, alternative targets such as competence and students’ early exposure and experience are also related to resilience.

Other studies focused on developing resilience curriculum, incorporating aspects such as psychological support, grading changes, and competence training. An author developed the HYMS CARE criteria to evaluate the needed components to create a resilience curriculum.^[60]^ The criteria were used as curriculum assessment tools to focus on several internal and external factors, such as students’ resourcefulness, teamwork, and both internal and external support. However, this study did not describe the effectiveness of such criteria in influencing resilience or other mental health problems. Other studies have found that incorporating a wellness program into the curriculum was associated with increased well-being. One group incorporated mental health services, seminars, and student-proposed wellness activities into the medical curriculum.^[65]^ Another group described comprehensive curricular changes through decreased contact time, a shift in grading systems, and extracurricular activities based on the Saint Louis University School of Medicine and found that this intervention significantly lowered rates of anxiety and depressive symptoms.^[15]^ It seemed promising that a curriculum focusing on students’ wellness and resilience would greatly help our future physicians. However, implementing curricular changes in the dynamic landscape of the clinical setting might present challenges.

## 4. Discussion

Our results have shown that many studies supported our hypothesis that resilience is essential to combat burnout in medical students. Although resilience interventions have been shown effective, there is considerable variation in their success, with competence-based interventions showing promise for long-term benefits. However, 1 main drawback of research focusing on resilience is the different definitions of resilience currently in use. For example, the American Psychological Association Dictionary of Psychology defines resilience as “the process and outcome of successfully adapting to difficult or challenging life experiences.”^[71]^ Another author defined resilience as “the ability to maintain the persistence of one’s orientation toward existential purposes.”^[26]^ Note that resilience was described as a process, an outcome, or an ability between these 2 definitions. However, resilience may exist along a spectrum with many forms that differ across life’s domains. Resilient coping styles were shown to dismiss the risk of stress and burnout and maintain functions in many populations, such as caregivers, patients, military personnel, and healthcare workers whose jobs require constant exposure to emotional stress. Students, children, and young adults also benefited from resilient coping styles.^[72–75]^ Although resilient coping styles can be equated with resilience, it might be debatable.

Resilience can also be described as consisting of several categories or domains. An attractive model of academic resilience was proposed by Martin and Marsh, defining resilience as the 5-C model consisting of confidence, coordination, control, composure, and commitment.^[31]^ Although there are other definitions of resilience, this model is suitable for explaining the rationale for interventions and how resilience might interact with the clinical learning environment. We argued that 3 of the model’s 5 components are especially important concerning medical students in clinical training. These are composure/low anxiety, confidence/self-efficacy, and control or a lack thereof. Nevertheless, most of the studies we explored in this article did not explain their working definition or contain different working definitions for resilience. Some studies define resilience as psychological endurance, while others emphasize clinical competence, resulting in different approaches to building resilience. This is also the basis for the low-quality evidence level concluded by a previous Cochrane review on resilience training.

### 4.1. Resilience as a buffer for burnout

Burnout is a well-documented problem experienced by medical students, particularly during clinical rotations. The transition from theoretical learning to hands-on patient care was a known risk factor and stressor. Our findings demonstrated that resilience serves as a protective factor against burnout, with higher resilience scores correlating with lower levels of burnout.^[28,47]^ However, the many definitions and measures of resilience hindered the development of specific interventions to increase resilience. Nonetheless, we can conclude that medical students who exhibit greater resilience are more likely to cope effectively with the stresses of clinical training, maintaining better psychological health and engagement in learning. However, we have to note that resilience alone may not prevent burnout. A study indicated that resilience was an effective buffer against physical, not psychological, demands.^[28]^ Conversely, other studies found that resilience mediated the positive effect of 2 psychological constructs: social support and empathy.^[44,50]^ A survey by Neufeld et al may explain the conflicting findings. They found that although resilience is important, student’s basic psychological were essential factors and, when considered in their model, effectively reduced the association between resilience and perceived stress.^[41]^ Therefore, although resilience is an essential buffer in reducing stress, this reduction is lower when the student’s basic psychological needs are already taken care of, explaining their protective ability against physical demands. Intriguingly, the authors identified one of the basic needs as competence, a trait shared with resilience according to several definitions.^[31]^ Therefore, the reduction of association might be due to the statistical correlation between resilience and basic psychological needs in the final model. Although currently, many unknown factors interact and influence resilience, the results from these studies have shown that resilience plays a vital role in decreasing the adverse effects of stress on students’ mental health and well-being. Therefore, since students with high resilience still experience significant stress during clinical rotation, interventions must be paired with other supportive interventions to primary students’ needs, such as competence and autonomy, to maximize its protective effects.

### 4.2. The effectiveness of resilience interventions

One of the most striking findings of our review is the various forms of resilience-building interventions with differing effectiveness. Psychological interventions, such as mindfulness and stress management workshops, are commonly used to enhance resilience. However, our review indicates that these interventions often provide limited long-term benefits, with some not reporting the benefits gained from such interventions.^[76]^ In contrast, interventions focusing on clinical competence showed a promising result with a more prolonged sense of empowerment and confidence.^[70]^ The Cochrane review by Kunzler et al (2020) also concluded that resilience training had a modest effect on reducing anxiety and stress among healthcare students, with the heterogeneity of interventions and short follow-up periods limiting the strength of the evidence.^[66]^

Several research studies in our review focus directly on shame and identify shame as a target for increasing resilience. The Emotional Speed Dating intervention helps students face the uncertainties of patient encounters.^[56]^ This approach can help them share their experiences, reducing loneliness. Although the effects of the intervention on resilience have not been validated, their theoretical basis is sound and addresses shame, a powerful emotion experienced by many students. Another example is the authors conducting shame seminars.^[57,58]^ Although they did not directly link shame with resilience, they described that a more constructive handling of shame might indirectly improve student resilience. Studies focusing on team dynamics also focus on addressing shame. At its core, the intervention aimed to normalize handling difficult emotions or, in another way, to deal with shame. A study by Houpy et al identified that students brave enough to discuss their errors and problems with peers have higher resilience scores.^[53]^ Their findings further corroborate that addressing shame by normalizing difficult emotions and acknowledging mistakes can increase student resilience and improve overall well-being.

Nevertheless, several major drawbacks are apparent from most of the studies. Most of these studies with novel interventions did not validate whether their intervention is effective at increasing resilience^[56–58]^ or has a short follow-up period.^[63,66]^ A study reported that students continue to utilize the knowledge gained from the “Active Resilience Training” program used in the study for at least 18 months after the end of their training.^[59]^ This suggests that resilience training can have lasting effects if designed to be practical and applicable to real-world challenges. However, studies suggested that resilience interventions are most effective when students can choose their participation. Mandatory programs may undermine this sense of control, potentially negating their benefits.^[61–63]^ According to Martin and Marsh, students’ need for autonomy is one of the core components of resilience^[31]^ and is perhaps one of the most elusive in clinical training. For students who have shown mastery, entrusting them with a professional activity will generate feelings of competence and autonomy,^[77]^ 2 components of resilience. However, we would like to note that supporting students’ autonomy does not mean sacrificing patient safety. Several authors have discussed balancing the need for patient safety and student autonomy.^[78,79]^ Ma reviewed the effectiveness of autonomy support by teachers, and they concluded that autonomy support increased students’ engagement and resilience. However, the studies the review was based on are not focused on medical students.^[80]^ Additionally, short online programs offering resilience training were shown to be effective while providing these students flexibility and might be a viable option instead of forcing mandatory interventions.^[67]^

On the other hand, interventions focused on clinical competence appear to offer more sustainable benefits. For example, Levy-Carrick et al (2019) found that training medical students in clinical skills, such as hemorrhage control, increased their competence and fostered resilience-related traits like self-efficacy and confidence.^[70]^ This suggests that resilience interventions that enhance students’ sense of mastery and control in clinical environments may be more effective than purely psychological approaches.^[30,70]^ Therefore, interventions need to focus on psychological needs and clinical competence. We argue that such training should also be done for medical students whose clerkship stage already exposes them to work-related stress.

Other intervention methods worth discussing tried to intervene at a more structural level concerning implementation at the curriculum level. A study by Heinen et al supports this approach.^[14]^ The authors identified that although coping skills and resilience modulate the stress response, these personal resources could not prevent the increased rate of anxiety, depression, and stress that the curriculum forces on medical students. Therefore, they concluded that a curricular approach must also be taken. This method seemed reasonable, especially considering that many, if not all, components of resilience can be improved through curricular changes. A study advocated mapping the determinants of resilience to the current curriculum, ensuring that the course aligns to increase resilience.^[24]^

### 4.3. Limitations of current studies and future directions

One of the critical limitations of our review is that many studies on resilience training are limited by short follow-up periods and a lack of standardized measures for resilience. Additionally, the heterogeneity of interventions complicates the ability to draw general conclusions about the best approaches. Future studies should aim to standardize resilience measures and explore the long-term efficacy of interventions that combine psychological support with clinical competence training. Additionally, the effects of competence training on resilience are still much less studied than other psychological interventions. Focusing on the whole picture instead of just focusing on the psychological aspects of resilience (i.e., stress reduction) may be more effective for students. Other factors, such as autonomy and the 2 components we have not touched (coordination and commitment), are also less researched. Although these components are arguably better intervened through curricular changes by allowing students to work towards the goal and to plan effective measures to achieve such goals. Therefore, further research is needed to explore the effectiveness of implementing curricular changes in increasing resilience and promoting students’ well-being.

## 5. Conclusion

Resilience is critical in reducing burnout among medical students, particularly during clinical rotations. While psychological interventions can help students cope, competence-based training is a promising alternative, offering sustainable resilience benefits. The medical curriculum should incorporate resilience training that balances psychological support with practical clinical skill development to better prepare students for the stresses of clinical practice. Our students need as much innovation in teaching and supporting resilience as possible to prepare them for the arduous journey in medicine.

## Acknowledgments

We want to thank Charissa Lazarus for providing input in the creation of this review. We also thank Maranatha Christian University for supporting article access for this review.

## Author contributions

**Conceptualization:** Nathanael Andry Mianto, Christian Edwin.

**Formal analysis:** Ardo Sanjaya, Nathanael Andry Mianto, Karen Regina Wijayanto.

**Investigation:** Ardo Sanjaya.

**Project administration:** Ardo Sanjaya, Karen Regina Wijayanto.

**Supervision:** Ardo Sanjaya, Christian Edwin.

**Validation:** Ardo Sanjaya, Christian Edwin.

**Writing – original draft:** Ardo Sanjaya, Karen Regina Wijayanto, Christian Edwin.

**Writing – review & editing:** Ardo Sanjaya, Nathanael Andry Mianto, Karen Regina Wijayanto, Christian Edwin.

## Supplementary Material


